# Transabdominal ultrasound for positive, negative, and equivocal ovarian and tubal torsion in girls

**DOI:** 10.1007/s10140-025-02399-2

**Published:** 2025-10-08

**Authors:** Julian Lopez-Rippe, Maria Camila Velez-Florez, Rosa Hwang, Wondwossen Lerebo, Gary Nace, Aaron E. Chen, J. Christopher Davis, Eron Friedlaender, Summer L. Kaplan

**Affiliations:** 1 Department of Radiology, Children’s Hospital of Philadelphia, 3401 Civic Center Blvd, Philadelphia, PA 19104 USA; 2 Department of Surgery, Children’s Hospital of Philadelphia, 3401 Civic Center Blvd, Philadelphia, PA 19104 USA; 3 Department of Pediatrics, Emergency Medicine, Children’s Hospital of Philadelphia, 3401 Civic Center Blvd, Philadelphia, PA 19104 USA; 4 Perelman School of Medicine, University of Pennsylvania, 3400 Spruce St, Philadelphia, PA 19104 USA

**Keywords:** Pediatric, Emergency, Adnexal torsion, Ovarian torsion, Tubal torsion, Ovary, Fallopian tube, Ultrasound, Equivocal

## Abstract

**Background:**

Adnexal torsion diagnosis in children relies heavily on ultrasound, but existing literature lacks standardization of technique and handling of equivocal results.

**Purpose:**

To assess the accuracy of transabdominal ultrasound (US) in diagnosing adnexal torsion in pediatric patients and evaluate optimal clinical decision-making for equivocal US reads.

**Materials and methods:**

Retrospective review of pelvic US exams and surgical data for girls aged 1–18 years from 2015 to 2019 at a pediatric quaternary care hospital. US reports were coded as positive, negative, or equivocal for torsion. Surgical findings were used to confirm final diagnosis. Sensitivity, specificity, positive predictive value (PPV), and negative predictive value (NPV) were calculated under various scenarios for handling equivocal reads.

**Results:**

This study included 4,396 ultrasound exams from 3,707 patients (median age, 15.2 years [IQR: 12.8–16.8]). Ovarian visualization rate was 97–98%. US was read as positive for torsion in 1% of adnexae, negative in 95%, and equivocal in 4%. Among 179 surgical cases, torsion was present in 52%. Sensitivity of US for torsion was highest (86.0%) when equivocal cases were considered positive (EqP), but PPV was lowest (29.9%) under this condition. Area under the ROC curve was greatest (0.8651) when equivocal US were counted as positive. US reads were more likely to be true positive for isolated ovarian torsion compared to when tubal torsion was present (*p* = 0.003).

**Conclusion:**

A standardized transabdominal US protocol yields high ovarian visualization rates, and treating equivocal reads as positive can optimize diagnostic accuracy when combined with clinical decision-making. US maybe be less accurate in detecting tubal torsion compared to isolated ovarian torsion, but this finding should be interpreted with caution given the small sample size. Overall, these results provide clinically relevant insights to guide management and future research in pediatric adnexal torsion.

**Supplementary Information:**

The online version contains supplementary material available at 10.1007/s10140-025-02399-2.

## Introduction

Adnexal torsion refers to the twisting of an ovary, fallopian tube, or both structures along their vascular pedicles, resulting in obstruction of venous outflow and arterial inflow. This compromises blood supply to the adnexa and can rapidly progress to necrosis if untreated. Most commonly the ovary is affected, but the fallopian tube may also torse, often in association with an underlying abnormality like a paratubal cyst [1]. Prompt diagnosis and surgical detorsion are crucial to preserve future ovarian function and fertility.

Adnexal torsion is a significant contributor to gynecological emergencies, affecting 2–7% of women who undergo surgical procedures due to acute pelvic pain [2]. Adnexal torsion can occur at any age, but most cases occur in the reproductive years, with annual incidence estimates ranging from 0.1 to 4.9 per 100,000 females [3, 4]. Rates are higher in adolescents and pregnant women [5]. Approximately 20% of cases occur in premenarchal girls, and 15% involve women with ovarian masses [6, 7]. Despite the frequency of adnexal torsion in girls, there is a paucity of imaging literature focused on this diagnosis in children. Ultrasound (US) is the first-line imaging modality at all ages, the existing literature often lacks description of US technique, which may affect diagnostic accuracy in children for whom transvaginal US is not routinely performed. In women, accurate sonographic diagnosis ranges from a reported 23–81%, while literature in girls reports rates of 21–92% [8–12].

Existing research often focuses on US exams validated by surgical results [8, 10, 13], but this case selection does not model diagnostic decision-making at the point of care, in which the vast majority of US exams will not be followed by surgery. It has been suggested that non-visualization of ovary in girls is sufficient to exclude torsion, but further research is needed to strengthen clinical decision-making around equivocal US cases, which are under-represented in the scientific literature. In our study, only a very small number of torsion cases presented with non-visualization, and these were associated with technical issues or anatomic factors that limited sonographic access. This highlights that while non-visualization generally lowers suspicion, it cannot fully exclude torsion and must be interpreted in the context of clinical findings. It is also unclear whether tubal torsion in girls poses unique diagnostic challenges compared with the more commonly described appearance of ovarian torsion.

This research aims to assess the accuracy of transabdominal ultrasound in diagnosing adnexal torsion in pediatric patients and evaluate optimal clinical decision-making for equivocal US reads through a large retrospective review of cases from a pediatric hospital over a five-year period.

## Materials and methods

This retrospective, low-risk study was deemed exempt by the institutional review board. The work was conducted at a 567-bed pediatric quaternary care hospital with approximately 100,000 emergency department (ED) visits per year. The Radiology Department has 24/7 in-house US technologist, MR technologist, and diagnostic radiology attending coverage with final reads. At our institution, US evaluation for adnexal torsion is conducted using transabdominal scanning through a full bladder. ED staff are expected to check bladder volume with a bladder scanner or point-of-care US before radiology US to improve visibility of the adnexa, ensuring patients have at least the following bladder volumes by age or developmental stage: 75 ml at ≤ 5-years old; 150 ml for 6-years old to menarche; 250 ml for post-menarchal patients. The clinical pathway for suspected adnexal torsion at our institution begins with transabdominal US, while pelvis MR is recommended as a secondary modality when further imaging is needed after a non-diagnostic or equivocal US. Transvaginal US is not routinely performed or offered in our pediatric ED. Use of imaging modalities may vary by ED or radiology physician preference and specific clinical considerations. Pediatric general surgeons staff clinical consults and any necessary surgery for adnexal torsion; no gynecology service is available.

### Study population

Our team retrospectively reviewed imaging and operative data for girls ranging in age from ≥ 1-year old to ≤ 18-years old between January 1, 2015 and December 31, 2019. Patient age at the time of US was recorded. After applying inclusion and exclusion criteria as described below, we matched patient medical record number and date of service to combine imaging and operative data.

#### Imaging data

Pelvic US exams performed in the ED for patients in the eligible age range were included. Exams were excluded if transvaginal US was performed, if pelvic US was not performed under the exam code for pelvic US, or if neither uterus nor ovaries were seen.

#### Operative data

For the time interval and age range of interest, surgical cases with the following Common Procedure Terminology (CPT) codes were identified: laparoscopic total oophorectomy (58661); laparoscopic biopsy or excision of ovarian mass (58662); laparoscopic ovarian cyst excision or marsupialization (58662); laparoscopic reduction of ovarian torsion (58679); salpingectomy (58700); ovarian teratoma resection/excision (58920); oopherocystectomy (ovarian cyst excision) (58925); ovarian mass resection, possible oophorectomy (58925); oopherectomy, partial (58940), oopherectomy, with or without salpingectomy (58940), oophoropexy, unilateral/bilateral (ovariopexy) (58999); ovary, torsion reduction (58999); laparoscopic aspiration of ovarian cyst (49322); and laparoscopic exploration/diagnostic (49320) with diagnosis associated with ovarian or adnexal pathology. Surgical cases were excluded if they were performed for genetic gynecological conditions or if no pre-operative ultrasound was performed on-site.

### Data coding

#### Imaging coding

Radiology reports for pelvic US were extracted from the radiology information systems. One research assistant (M.C.V.F. 4 years clinical emergency medicine, 2 years research experience) and one pediatric radiologist (S.L.K. 7 years experience) reviewed all radiology reports and coded each adnexa individually for whether the ovary was visualized and whether oophorectomy was reported. Each adnexa was individually coded as positive, negative, or equivocal for adnexal torsion based on the radiology report using the following guidelines:

- Positive reports were defined by the following terms or like terms in the radiology report impression: “concerning for” torsion; “suspicious for” torsion; “consistent with” torsion; “suggestive of” torsion; “torsion is present”.
- Negative reports were defined by the following terms in the radiology report impression: “no torsion”; “no evidence of torsion”; “normal female pelvis ultrasound.”
- Equivocal reports were defined from the complete radiology report, including the final impression as documented by the interpreting radiologist, when terms such as: “evaluation equivocal”; “indeterminate”; “evaluation could not be obtained”; “suboptimal visualization”; “torsion cannot be excluded”; “further evaluation recommended”.
  - Reasons for equivocal exams were further categorized as:
    - Ovary not visualized.
    - Cyst or mass complicating evaluation.
    - Doppler signal insufficient.
    - Ovary size or position of concern, or presence of tubal fluid.

Images were not reviewed during coding due to the large number of exams enrolled in the study and to simulate clinical conditions in which patient care decisions are made based on the radiology report.

#### Operative coding

Operative reports were extracted from the electronic health record (EHR) and were reviewed by a surgical research coordinator (R.H. 2 years experience), who coded for presence of ovarian torsion, tubal torsion, or both. Other adnexal abnormalities described intraoperatively were also recorded. A pediatric surgeon (G.N. 10 years experience) validated the accuracy and completeness of extracted operative data through an independent second review.

## Definitions

Ultrasound reports were assessed using contingency tables with each adnexa coded individually. This adnexum-level approach was chosen because radiology reports are structured per ovary/tube and equivocal findings frequently involve only one side. No patients in our cohort had bilateral acute adnexal torsion, so patient-level analyses would not have altered the overall findings.

- **True positive** (TP) ultrasound for torsion was defined as radiology read positive for torsion and surgery positive for torsion.
- **False positive** (FP) was radiology read positive for torsion and either surgery negative for torsion or no surgery.
- **True negative** (TN) was defined as radiology read negative for torsion and surgery negative for torsion or no surgery.
- **False negative** (FN) was defined as radiology read negative for torsion and surgery positive for torsion.
- Ultrasound exams categorized as equivocal were defined as positive (EqP, equivocal considered positive) if surgery showed torsion or negative (EqN, equivocal considered negative) if surgery showed no torsion or if no surgery was performed.

### Statistical analysis

Statistical analysis was performed using STATA software (Stata Statistical Software: Release 18. College Station, TX: StataCorp LLC.) Visualization of the ovaries, reports read as positive, negative, or equivocal for torsion, and reasons for equivocal reads were summarized using frequency and percentage. Age of patients was summarized as median and interquartile range Diagnostic yield of US for adnexal torsion was performed by comparing radiology reads to surgical results or non-surgical management. Sensitivity, specificity, positive predictive value (PPV), and negative predictive value (NPV), were modeled for the clinical decision-making scenarios:

- Equivocal reads omitted.
- Equivocal reads all considered positive.
- Equivocal reads all considered negative.
- Equivocal reads due to mass or cyst considered positive; equivocal reads due to other reasons considered negative.

We also assessed whether the presence of tubal torsion made US interpretation more challenging. For cases with surgically positive torsion, we used frequency tables to characterize the number of adnexae read as positive, negative, or equivocal for torsion when: (1) only ovarian torsion was present; (2) tubal torsion was present alone; or (3) tubal torsion was present together with ovarian torsion. Pearson Chi-squared test assessed whether differences were significant. Statistical significance was declared at p-value ≤ 0.05 for all analyses.

## Results

**Fig. 1:**
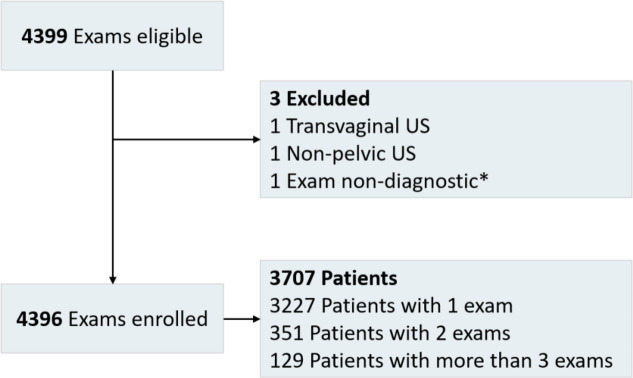
Enrollment flowchart *non-diagnostic if uterus and ovaries both not seen

Imaging data collection identified 4,399 eligible US exams, of which 4,396 US exams were enrolled, including 3,707 patients, (Fig. 1). Patient age at the time of exam was median 15.2 years (IQR: 12.8–16.8).

The right ovary was visualized in 98% of exams (*N* = 4285/4396) and the left ovary was visualized in 97% (*N* = 4231/4396), while both ovaries were visualized in 95% (*N* = 4192/4396). Oophorectomy was responsible for non-visualization in 21 adnexae. US was read as positive for torsion in 1% of adnexae (*N* = 96/8792), negative for torsion in 95% (*N* = 8338/8792), and equivocal in 4% (*N* = 358/8792). Reasons for equivocal reads included: ovary not seen (57%, *N* = 204/358); cyst/mass limiting evaluation (27%, *N* = 98/358); Doppler signal inadequate (8%, *N* = 29/358); concern for ovary size, position, or presence of tubal fluid in (8%, *N* = 27).

Adnexal surgery was performed 179 times following ultrasound. Torsion was present in 52% of surgical cases (*N* = 93/179), of which 54% were ovary alone (*N* = 50/93), 31% ovary and tube (*N* = 29/93), and 15% tube alone (*N* = 14/93). The right adnexal structures were torsed in 59% (*N* = 55/93), while left structures were torsed in 41% (*N* = 38/93) (*p* = 0.044). Surgical cases without torsion had findings of ovarian cyst (34%, *N* = 61), ovarian mass (7%, *N* = 13), paraovarian/paratubal cyst (7%, *N* = 12), tubo-ovarian abscess/pelvic inflammatory disease (2%, *N* = 4), and endometriosis (< 1%, *N* = 1). Some cases had more than one non-torsion finding. Only three surgical cases had no adnexal pathology. One of these was positive for appendicitis, one had a normal radiology read but a history of repeated adnexal torsion, and the third had an US read of concern for torsion/detorsion based on midline position of ovary.

The majority of adnexae assessed were read as negative for torsion and did not go to surgery (TN, *N* = 8131/8792) (Table 1). US read as positive for torsion were surgically positive 61% of the time (TP, *N* = 57/93). Among adnexae read as equivocal for torsion, the highest percentage of cases going to surgery occurred in the presence of a cyst or mass (*N* = 45/98, 45%). When ovary was not seen, treatment was non-surgical in 95% of cases (*N* = 194/204). Four non-visualized ovaries were surgically positive for torsion. Detailed review of these four cases showed that non-visualization was related to patient body habitus, bowel gas, or unspecified technical factors. In each case, subsequent MRI confirmed torsion, leading to surgery. Cases equivocal due to inadequate Doppler signal never went to surgery.

**Table 1:** Contingency table for ultrasound reports

Radiology Report Result​	Surgery​ Positive Torsion, *N*	Surgery Negative Torsion​, *N*	No Surgery, *N*​	Total​ Reports, *N*
**Positive torsion**​	**TP**: 57	**FP**: 24	**FP**: 15	96
**Negative torsion**​	**FN**: 21	**TN**: 196	**TN**: 8121	8338
**Equivocal**​	**EqP**: 15	**EqN**: 45​	**EqN**: 298​	358
**Total outcomes**	**93**	**265**	**8434**	8792
***Reasons for equivocal***				
*Ovary not seen*	4	6	194	204
*Cyst/mass*	9	36	53	98
*Doppler inadequate*	0	0	29	29
*Size*,* position*,* or tubal fluid*	2	3	22	27

TP = true positive. FP = false positive. FN = false negative. TN = true negative. EqP = equivocal counted as positive. EqN = equivocal counted as negative

**Fig. 2:**
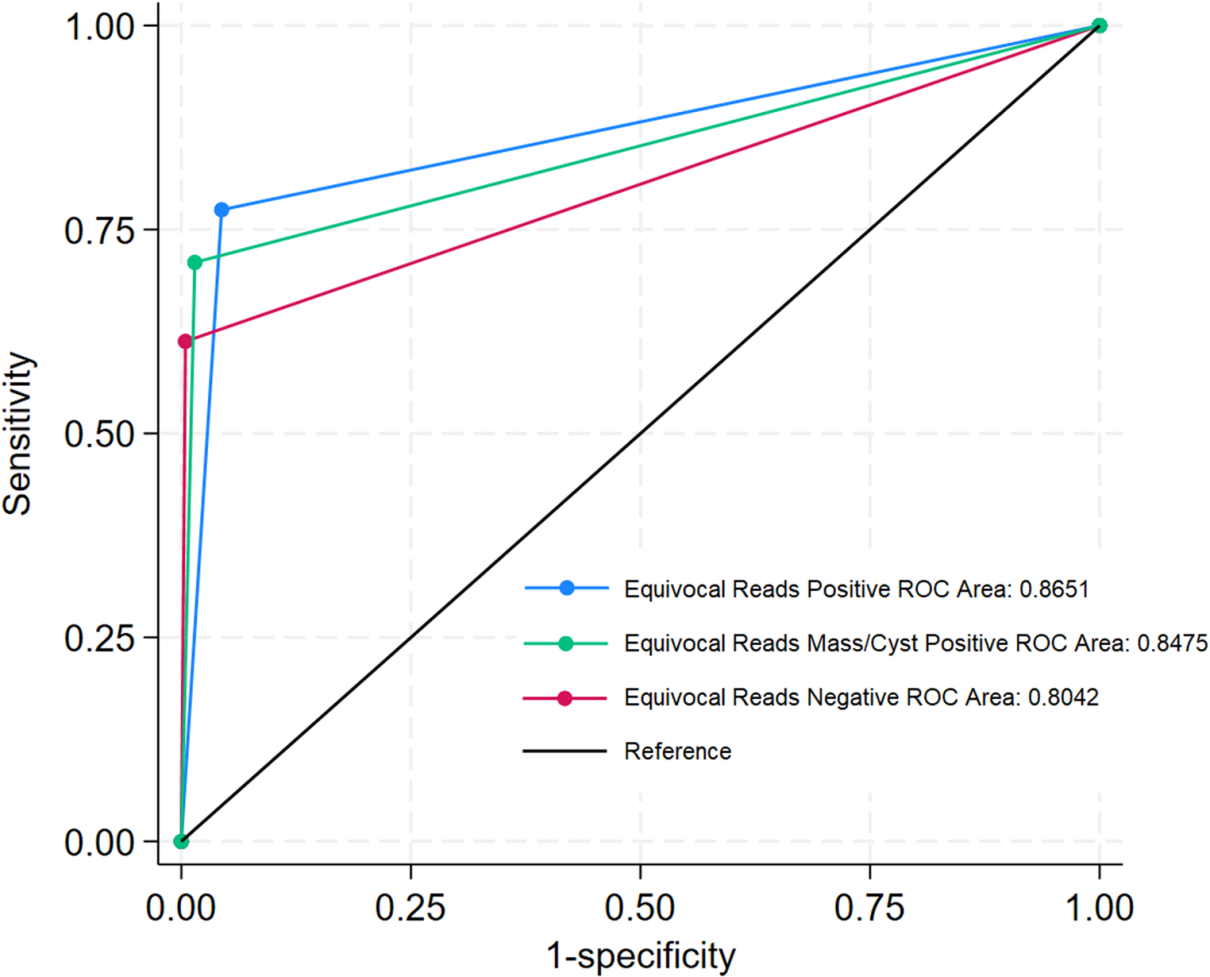
Receiver Operating Characteristic (ROC) Curves for Equivocal Radiology Reads

Sensitivity of US for torsion was highest when equivocal cases were considered positive (Table 2). However, PPV was lowest under this condition. Due to the high number of TN US exams, namely those that did not proceed to surgery, the specificity and NPV of US for torsion did not change much across different conditions. Receiver operating characteristic (ROC) showed greatest area under the curve (AUC) when equivocal US were counted as positive (p-value = 0.005) Considering equivocal cases as positive only when cyst/mass was present did not significantly improve the test characteristics. (Fig. 2).

**Table 2:** Clinical decision scenarios with sensitivity, specificity, positive predictive value, and negative predictive value

Scenario​	Sensitivity​	Specificity​	PPV​	NPV​
*Equivocal cases​ omitted*	57/78 (73.1%; CI 61.8–82.5%)​	8317/8356 (99.5%; CI 99.4–99.7%)	57/96 (59.4%; CI 48.9–69.3%)​	8317/8338 (99.7%; CI 99.6–99.8%)​
*Equivocal cases considered positive​*	72/93 (77.4%; CI 67.6–85.4%)	8317/8699 (95.6%; CI 95.2–96.0%)​	72/454 (15.9%; CI 12.6–19.6%)​	8317/8338 (99.7%; CI 99.6–99.8%)​
*Equivocal cases considered negative​*	57/93 (61.3%; CI 50.6–71.2%)	8660/8699 (99.6%; CI 99.4–99.7%)​	57/96 (59.4%; CI 48.9–69.3%)​	8660/8696 (99.6%; CI 99.4–99.7%)​
*Equivocal due to cyst/mass lesions considered positive​*	66/93 (71.0%; CI 60.6–79.9%)​	8571/8699 (98.5%; CI 98.3–98.8%)​	66/194 (34.0%; 27.4–41.2%)​	8571/8598 (99.7%; 99.5–99.8%)

PPV = positive predictive value. NPV = negative predictive value

**Fig. 3:**
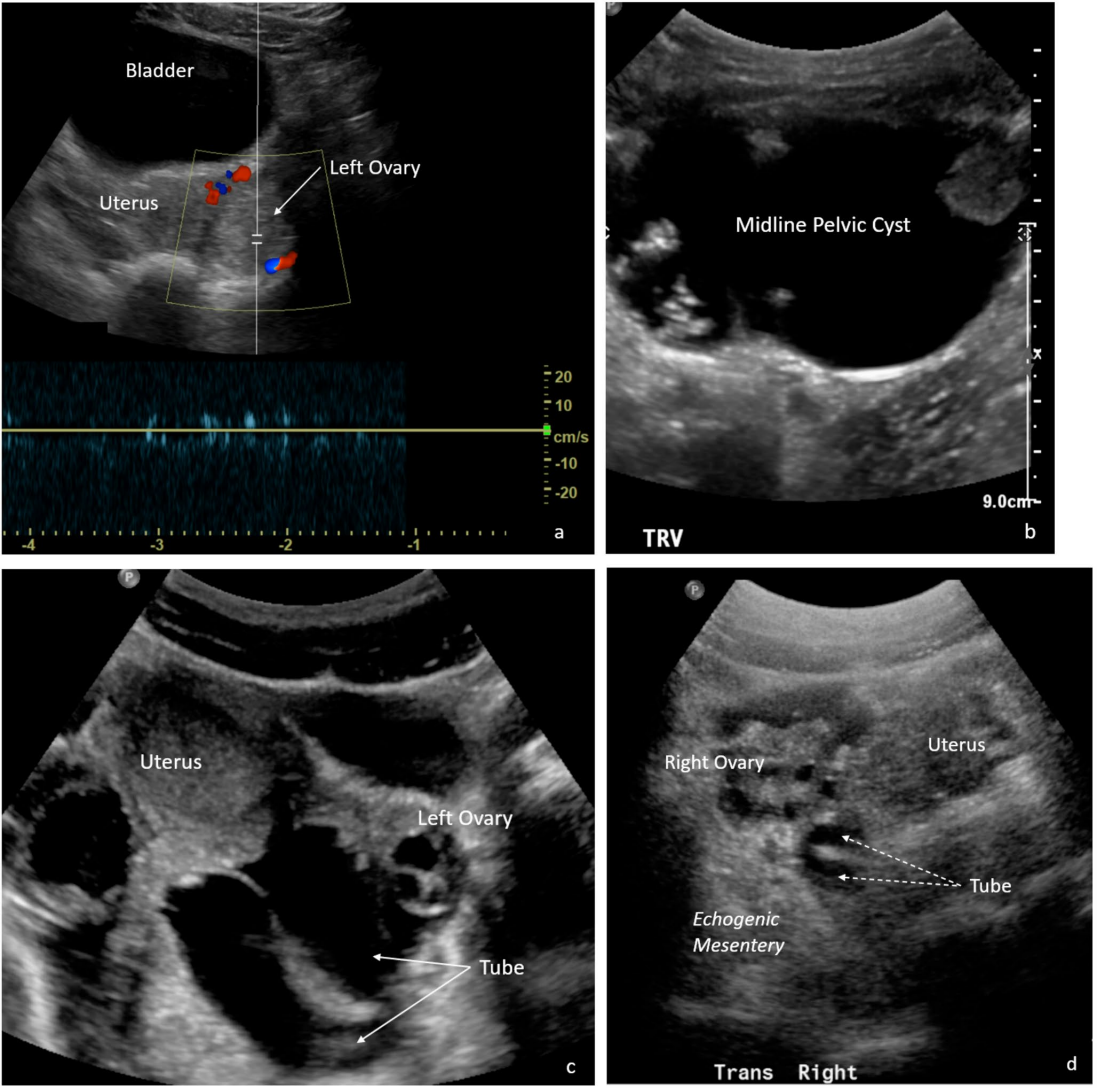
**False positive** ultrasound (US) in a 17-year-old girl with left pelvic pain shows a large left ovary with decreased to absent spectral Doppler signal (**a**); at surgery, this was a hemorrhagic cyst. **Equivocal** US in a 9-year-old girl with concern for abdominal mass shows an 8.4 cm complex adnexal cyst/mass in the pelvic midline, with left ovary not visualized (**b**); at surgery this was a left ovarian mass without torsion, which on pathology examination was a mature cystic teratoma. **True positive** US in a 13-year-old girl with left pelvic pain shows a fluid-filled tubular left adnexal structure (arrows) adjacent to a normal left ovary (**c**); at surgery, this was tubal torsion. **False negative** US in a 12-year-old girl with right pelvic pain was noted to have echogenic mesentery and a normal right ovary without torsion (**d**); at surgery, this was identified as a tubal torsion with a completely necrotic tube

Among surgical cases, US reads were more likely to be TP when only ovary was torsed and were more likely to be equivocal or FN when tubal torsion was present (*p* = 0.003) The highest percentage of false negative US reads occurred when the tube only was torsed. (Table 3; Fig. 3).

**Table 3:** Accuracy of US results among cases with surgical results of ovary-only torsion compared with presence of tubal torsion

US Report	Surgical results			
	Ovary only	Any tubal torsion (tube only or tube + ovary)	Tube only	Tube + ovary
US positive for torsion (TP)	36 (72%)	21 (48%)	5 (36%)	16 (55%)
US negative for torsion​ (FN)	10 (20%)	11 (26%)	8 (57%)	3 (10%)
US equivocal for torsion​	4 (8%)	11 (26%)	1 (7%)	10 (35%)
Total	50 (100%)	43 (100%)	14 (100%)	29 (100%)

TP = true positive. FN = false negative

## Discussion

Our review of US diagnosis for adnexal torsion in girls revealed new information about how we can best assess for torsion. We show a high rate of ovarian visualization (97–98%) using a standardized transabdominal US approach with age-dependent predetermined bladder volumes. Considering both surgical and non-surgical cases, we found that treating equivocal reads as positive was optimal (AUC = 0.8651), though equivocal reads due to inadequate Doppler signal never proceeded to surgery in our 5-year cohort. US reads showed greater accuracy with isolated ovarian torsion compared to cases involving tubal torsion (*p* = 0.003).

Current pediatric adnexal torsion literature lacks consistency in reporting ultrasound techniques and visualization rates. While transabdominal ultrasound is commonly mentioned, detailed scanning protocols are often omitted [10, 11, 14]. Visualization rates aren’t consistently reported across studies, making standardized benchmarking difficult. For instance, a 2007 study reported visualizing the affected ovary in 100% of torsion cases, suggesting high visibility when torsion is present. However, this may not reflect the general visibility of ovaries in all suspected cases [15]. Alternatively, a 2019 study found that ovaries were initially not visualized in 97.7% of cases with a non-distended bladder [12]. Our findings show that standardized transabdominal ultrasound protocol with full bladder achieves consistently high visualization rates, enhancing diagnostic validity. This underscores the need for structured, reproducible scanning methodology and standardized reporting templates in future studies, which would address current gaps in the literature and allow more meaningful comparison of diagnostic accuracy across institutions. Reporting templates should, at minimum, capture bladder volume at scan, method of ovarian visualization, Doppler technique/adequacy, and the presence of adnexal masses or free/tubal fluid.

Ultrasound diagnosis of ovarian torsion reports wide ranges for sensitivity (23–92%), specificity (53–99%), positive predictive value (19–82%), and negative predictive value (79-99.7%) [8–12]. This variation reflects differences in study designs, populations, and diagnostic criteria. Most studies lack consistent scanning technique descriptions, varying in transducer type, Doppler methods, and positive finding criteria [9, 10, 16]. Most studies included only surgically confirmed cases of torsion, which may lead to overestimation of diagnostic accuracy due to verification bias [8, 10, 13]. Some included clinically suspected cases with follow-up, though less frequently [12, 17]. These inconsistencies highlight the need for standardized reporting in pediatric adnexal torsion studies. In our study, both surgical and non-surgical cases were considered to determine the diagnostic characteristics of ultrasound. This approach combined with consistency in the ultrasound technique, more closely models the clinically relevant diagnostic characteristics of ultrasound evaluation for pelvic pain in girls.

Literature rarely explicitly discusses handling equivocal ultrasound reads in pediatric ovarian/adnexal torsion diagnosis. Most studies focus on patients with surgically confirmed diagnoses, potentially introducing selection bias by excluding cases that were equivocal on imaging but did not proceed to surgery. For example, previous work includes only patients who underwent surgery or had extended clinical/radiological follow-up [11, 18, 19]. This may overestimate diagnostic accuracy by excluding ambiguous cases. Studies generally didn’t explicitly analyze equivocal readings, instead forcing binary torsion classifications. Common reasons for equivocal reads included hemorrhagic cysts mimicking torsion, large masses obscuring normal ovarian tissue, and unreliable Doppler findings [11, 17–19]. However, the exact frequency of these issues was not consistently reported across studies. While these studies help us understand how to better search for findings associated with torsion, they leave gaps in our knowledge of the diagnostic value of US for adnexal torsion.

We modeled three scenarios for equivocal ultrasound interpretations and found optimal test characteristics when all equivocal cases were treated as positive; performance that did not improve when only cases with cysts or masses (previously associated with torsion) were considered positive. This approach achieved highest sensitivity and ROC performance but reduced PPV, potentially increasing unnecessary surgeries. However, equivocal interpretations comprised only 4% of examinations, mostly involving non-visualized ovaries that rarely led to surgery, limiting actual false-positive burden despite statistical PPV reduction. In clinical practice, ultrasound findings integrate with patient presentation, labs, and surgical consultation rather than being interpreted alone. Patients with equivocal findings but low clinical suspicion undergo observation or additional imaging like MRI, while high-suspicion cases proceed to surgery. This multidisciplinary approach enables equivocal reads to serve as a torsion detection safety net while clinical integration prevents unnecessary operations. Ultrasound diagnosis of adnexal torsion primarily focuses on ovarian torsion, with limited attention given to differentiating between ovarian and isolated tubal torsion. Feng et al. explicitly examined both types, reporting 94 cases (86%) of ovarian torsion and 15 cases (14%) of isolated tubal torsion in their study [19]. However, they did not differentiate the value of US for diagnosing ovarian versus tubal torsion. There are no studies that provide separate sensitivity or specificity values for diagnosing ovarian versus tubal torsion. In our study, we found a 59% true positive rate for adnexal torsion overall, but we found greater accuracy in US reads when only ovarian torsion was present. The US features associated with tubal torsion are less commonly recognized or taught, which may lead to lower rates of suspecting and correctly diagnosing tubal torsion. Our findings suggest that US may be less accurate in detecting tubal torsion compared to isolated ovarian torsion; however, given the small number of isolated tubal torsion cases in our cohort (*n* = 14), these results should be interpreted with caution and considered hypothesis-generating.

As a retrospective study, there were limits to our data collection due to limits in the existing documentation. We did not perform a secondary review of images given the large number of exams in our study (*N* = 4396). However, findings from our study do point to areas where image review with a more limited focus, such as a better understanding of the US features of tubal torsion, would be useful. We studied US only, whereas some of these patients may have proceeded to additional imaging to aid in clinical decision-making. Additional imaging may have changed the likelihood of proceeding to surgery. Given torsion’s dynamic nature, some false positive or negative US results may have been accurate at scan time, with subsequent detorsion or torsion before surgery. Another important limitation is potential verification bias, as the majority of adnexa (*N* = 8,131) that did not undergo surgery were assumed to be true negatives. This may overestimate specificity and NPV, since cases of resolved torsion, atypical presentations, or inadequate follow-up could be misclassified. While inherent to retrospective designs relying on real-world clinical pathways, this highlights the need for cautious interpretation of performance metrics and prospective studies with standardized follow-up to quantify misclassification effects on diagnostic accuracy. In addition, diagnoses were based on final radiology reports without image re-review, which limits assessment of inter-reader variability and the accuracy of equivocal labeling. Nevertheless, this approach reflects real-world decision-making based on finalized radiology reports, and future work with systematic image re-review would provide additional insight into variability and diagnostic accuracy. Finally, analyses were performed at the adnexum level rather than the patient level; although no bilateral torsion cases occurred, this approach may modestly skew diagnostic metrics but reflects real-world reporting and decision-making.

## Conclusion

Our findings highlight the importance of standardized transabdominal US protocols, which yielded high ovarian visualization rates and showed that treating equivocal reads as positive can improve diagnostic accuracy when integrated with clinical decision-making. US may be less accurate in detecting tubal torsion compared to isolated ovarian torsion, but this observation is based on a small sample and should be interpreted with caution. Overall, these results provide clinically relevant insights to improve diagnosis, guide management, and inform future research in pediatric adnexal torsion.

## Supplementary Information

Below is the link to the electronic supplementary material.

Supplementary Material 1

## Data Availability

The authors declare that they had full access to all of the data in this study and take complete responsibility for the integrity of the data and the accuracy of the data analysis.
