# Supplementary material for: Transabdominal ultrasound for positive, negative, and equivocal ovarian and tubal torsion in girls
Source: Emerg Radiol. 2025 Oct 8;33(1):33–41. doi: 10.1007/s10140-025-02399-2 (PMC12901135; doi:10.1007/s10140-025-02399-2)
Supplement: Supplementary file 1 — Supplementary Material 1 [file 10140_2025_2399_MOESM1_ESM.docx]

**Appendix 1**

**Definitions of Report Categories Based on Radiology Report Impressions**

| **Category** | **Definition** | **Example Terms** |
| --- | --- | --- |
| **Positive Reports** | Defined by terms indicating a high likelihood of ovarian torsion. | "Concerning for torsion," "Suspicious for torsion," "Consistent with torsion," "Suggestive of torsion," "Torsion is present." |
| **Negative Reports** | Defined by terms indicating the absence of ovarian torsion or a normal study. | "No torsion," "No evidence of torsion," "Normal female pelvis ultrasound." |
| **Equivocal Reports** | Defined by terms indicating uncertainty or limited diagnostic confidence. | "Evaluation equivocal," "Indeterminate," "Evaluation could not be obtained," "Suboptimal visualization," "Torsion cannot be excluded," "Further evaluation recommended." |

**Appendix 2**

**Surgical Cases and Associated Common Procedure Terminology (CPT) Codes**

| **Procedure** | **CPT Code** |
| --- | --- |
| Laparoscopic total oophorectomy | 58661 |
| Laparoscopic biopsy or excision of ovarian mass | 58662 |
| Laparoscopic ovarian cyst excision or marsupialization | 58662 |
| Laparoscopic reduction of ovarian torsion | 58679 |
| Salpingectomy | 58700 |
| Ovarian teratoma resection/excision | 58920 |
| Oophorocystectomy (ovarian cyst excision) | 58925 |
| Ovarian mass resection, possible oophorectomy | 58925 |
| Oophorectomy, partial | 58940 |
| Oophorectomy, with or without salpingectomy | 58940 |
| Oophoropexy, unilateral/bilateral (ovariopexy) | 58999 |
| Ovary, torsion reduction | 58999 |
| Laparoscopic aspiration of ovarian cyst | 49322 |
| Laparoscopic exploration/diagnostic | 49320 |
